# Characterization of drug resistance associated genetic polymorphisms among *Plasmodium falciparum* field isolates in Ujjain, Madhya Pradesh, India

**DOI:** 10.1186/1475-2875-13-182

**Published:** 2014-05-15

**Authors:** Ashish Pathak, Andreas Mårtensson, Sudhir Gawariker, Jagdish Mandliya, Ashish Sharma, Vishal Diwan, Johan Ursing

**Affiliations:** 1 Department of Pediatrics, R D Gardi Medical College, Surasa, Ujjain 456010, India; 2 Department of Women and Children’s Health, International Maternal and Child Health Unit, Uppsala University, Uppsala SE 751 85, Sweden; 3 Department of Public Health Sciences (Global Health/IHCAR), Karolinska Institutet, Tomtebodavägen 18A, Stockholm SE 171 77, Sweden; 4 Malaria Research, Department of Medicine Solna, Karolinska Institutet, Stockholm, Sweden; 5 Department of Medicine, R D Gardi Medical College, Surasa, Ujjain 456010, India; 6 Public Health & Environment in R D Gardi Medical College, Ujjain, India; 7 Centre for Clinical Research, Sörmland County Council, Sörmland, Sweden

**Keywords:** *Plasmodium falciparum*, Artesunate sulphadoxine-pyrimethamine, Chloroquine, Resistance, *pfcrt*, *pfmdr1*, *pfdhfr*, *pfdhps*, *pfnhe1*

## Abstract

**Background:**

Since 2011, artesunate + sulphadoxine-pyrimethamine (ASP), instead of chloroquine, has been recommended for treatment of uncomplicated malaria in India. In Ujjain, central India, with an annual parasite index <0.1, the prevalence of drug-resistant *Plasmodium falciparum* is unknown. In other parts of India chloroquine and sulphadoxine-pyrimethamine-resistant *P. falciparum* is prevalent. The aim of this study was to determine the prevalence of anti-malarial drug resistance-associated genetic polymorphisms in *P. falciparum* collected in Ujjain in 2009 and 2010, prior to the introduction of ASP.

**Methods:**

Blood samples from 87 patients with *P. falciparum* mono-infection verified by microscopy were collected on filter-paper at all nine major pathology laboratories in Ujjain city. Codons *Pfcrt* 72–76, *pfmdr1* 1034–1246, *pfdhfr* 16–185, *pfdhps* 436–632 and *pfnhe1* ms4760 haplotypes were identified by sequencing. *Pfcrt* K76T and *pfmdr1* N86Y were identified by restriction fragment length polymorphism, and *pfmdr1* gene copy number by real-time PCR.

**Results:**

Sulphadoxine-pyrimethamine resistance-associated *pfdhfr* 108 N and 59R alleles were found in 75/78 (96%) and 70/78 (90%) samples, respectively, and *pfdhps* 437G was found in 7/77 (9%) samples. Double mutant *pfdhfr* 59R + 108 N were found in 62/76 (82%) samples. Triple mutant *pfdhfr* 59R + 108 N and *pfdhps* 437G were found in 6/76 (8%) samples. Chloroquine-resistance-associated *pfcrt* 76 T was found in 82/87 (94%). The *pfcrt* 72–76 haplotypes found were: 80/84 (95%) SVMNT, 3/84 (4%) CVMNK and 1/84 (1%) CVMNT. *Pfmdr1* N86 and 86Y were identified in 70/83 (84%) and 13/83 (16%) samples, respectively. *Pfmdr1* S1034 + N1042 + D1246 were identified together in 70/72 (97%) of successfully sequenced samples. One *pfmdr1* gene copy was found in 74/75 (99%) successfully amplified samples.

**Conclusion:**

This is the first characterization of key anti-malarial drug resistance-associated genetic markers among *P. falciparum* collected in Ujjain, Madhya Pradesh, India. The results indicate that the efficacy of standard dose chloroquine at the time of the study was likely to be poor, whereas ASP was likely to be efficacious, supporting the changed drug treatment policy. However, *P. falciparum* with reduced susceptibility to sulphadoxine-pyrimethamine is highly prevalent, highlighting the need for continuous surveillance of ASP efficacy in the study area.

## Background

Approximately 85% of India’s population of 1.2 billion people live in malarious areas and transmission intensities vary from unstable to hyperendemic [1,2]. In 2012 there were 1.3 million reported malaria cases in India, although the true burden is probably considerably higher [2,3]. Furthermore, the proportion of the more virulent *Plasmodium falciparum* has increased in recent years and now accounts for 49% of the total malaria burden [2,4]. Since 2011 the Directorate of National Vector Born Disease Control Programme recommends that verified uncomplicated *P. falciparum* malaria and severe malaria should be treated with artesunate plus sulphadoxine-pyrimethmine (ASP) and quinine, respectively. Chloroquine (CQ) was the official first line drug prior to 2011. However, in areas with known CQ resistance, sulphadoxine-pyrimethmine (SP), between 1982 and 2005 and from 2005 ASP were recommended for treatment of uncomplicated malaria instead of CQ [4]. High grade SP resistance is widespread in parts of Southeast Asia and artemisinin resistance is developing along the Thai-Cambodia and Thai-Myanmar borders [5-7]. Furthermore, the PCR-corrected day 42 ASP failure rate was 9.5% (5/53) in a recent study from West Bengal [8]. There is thus a need to monitor AS-SP efficacy throughout India.

SP resistance in *P. falciparum* has been linked to single nucleotide polymorphisms (SNPs) A16V, C50R, N51I, C59R, S108N/T and I164L in dihydrofolate reductase (*pfdhfr*) and SNPs S436A/F, A437G, K540E, A581G and A613S in dihydropteroate synthase (*pfdhps*) [9-14]. Accumulation of SNPs typically starting with S108N leads to gradual reduction of susceptibility to SP and increased risk of treatment failure [15]. The quintuple *pfdhfr* 51I + 59R + 108N and *pfdhps* 437G + 540E haplotype is highly predictive of SP treatment failure in Africa [16]. In line with this, ASP treatment failures in West Bengal occurred in patients with *pfdhfr* 51I + 59R + 108N and *pfdhps* 437G or 436A + 540E [8]. These SNP combinations have also been reported from Odisha (eastern India) and the triple *pfdhfr* 59R + 108N + 164L haplotype has been reported from the northeastern state of Assam [17,18]. Moreover, quadruple *pfdhfr* 51I + 59R + 108N + 164L and *pfdhps* with three or four resistance-associated SNP combinations are common on the Andaman and Nicobar islands (Bay of Bengal) [19-21].

Resistance to CQ has developed in only five geographical locations but has spread from these points of origin to encompass most of the malaria endemic world [7,22]. A K76T SNP in the *P. falciparum* CQ resistance transporter gene (*pfcrt*) is essential for CQ resistance and this has been found at high frequencies throughout India [23-27]. Typically 76T is found together with additional SNPs creating specific *pfcrt* 72–76 haplotypes that indicate the origin of CQ resistance [22]. The *pfcrt* 72–76 haplotypes SVMNT from Papua New Guinea and CVIET from Southeast Asia are predominant in India [17,28].

In addition to these well-characterized, resistance-causing SNPs, *P. falciparum* multidrug resistance gene (*pfmdr1*) and sodium/hydrogen exchanger gene (*pfnhe1*) have been associated with anti-malarial drug resistance: *Pfmdr1* N86Y, S1034C, N1042D and D1246Y have been suggested to modulate levels of CQ resistance [29,30]. Increased *pfmdr1* copy number has been linked to reduced susceptibility to artemisinin derivatives, lumefantrine, piperaquine and mefloquine as well as artesunate + mefloquine treatment failure [31-34]. *Pfmdr1* N86 and *pfcrt* K76 have been linked to reduced lumefantrine susceptibility [35-37]. *Pfmdr1* S1034C, N1042D and D1246Y, >1 DNNND repeats and one DDNHNDNHNND repeat in the coding microsatellite ms4760 of *pfnhe1* have been linked to reduced quinine sensitivity [29,38,39].

Monitoring the frequency of *P. falciparum* resistance-associated genetic polymorphisms thus has the potential to identify declining susceptibility to a number of anti-malarial drugs. In Ujjain district located in Madhya Pradesh, central India, CQ was used until 2011 when ASP was introduced but no data on drug susceptibility of *P. falciparum* in the area exists. This study therefore analysed key anti-malarial resistance-associated genetic markers prior to the introduction of ASP for the first time. The report provides baseline data indicating a probable high efficacy of ASP and facilitates future monitoring of changing prevalence of resistance-associated genetic polymorphisms in this region as well as for comparisons with neighbouring areas.

## Methods

### Study site and period

Ujjain district is located in the western part of Madhya Pradesh, central India. The population of the district is 1.9 million as per 2011 Census [40]. The climate is tropical and transmission may occur throughout the year provided the relative humidity levels support the vector survival. Ujjain district has low transmission of malaria with annual parasite index (API) <0.1 in 2010 [41]. Peak malaria transmission occurs during the warm and humid months of July to September. Data collection was, therefore, done from June to October in 2009 and 2010.

### Recruitment of patients and sample collection

Samples and data were collected by the nine major pathology laboratories located in Ujjain city, Madhya Pradesh, India. Individuals or groups of pathologists with a postgraduate degree in pathology reported the results from participating laboratories. All laboratories used microscopical examination of peripheral blood smear for the diagnosis of *P. falciparum* malaria. The only inclusion criterion was microscopically verified malaria. For all patients that were smear positive for malaria, a drop of blood was put onto filter papers (Whatman™ 3MM). The filter papers were labelled with the patient’s age and sex, dried and then placed inside individual sealed plastic bags.

### Sample storage, DNA extraction

Filter papers were stored at room temperature. DNA was extracted using QIAamp Blood Mini Kit (QIAgen Biosciences, Germantown, MD, USA) according to the manufacturer’s instructions. Extracted DNA was stored at −20°C until use.

### Molecular analyses

*Pfcrt* 72–76, *pfmdr1* 1034–1246, *pfdhfr* 16–185, *pfdhps* 436–632 and *pfnhe1* ms4760 haplotypes were identified by PCR amplification followed by sequencing using previously described PCR protocols [23,37,42-44]. The Sequencher™ software version 4.6 (Gene Codes Corporation, Ann Arbor, MI, USA) was used for sequencing analysis. The *P. falciparum* 3D7 clone sequences obtained from NCBI database was used as references for *pfcrt* (Accession no NC_004328), *pfmdr1* (Accession no XM_001351751.1), *pfdhfr* (Accession no XM_001351443.1) and *pfdhps* (Accession no XM_001349382.1). *Pfnhe1* ms4760 sequences were compared with previously described isolates and clones.

In addition, previously described multiplex PCR-RFLP (restriction fragment length polymorphism) methods were used to identify *pfcrt* K76T and *pfmdr1* N86Y SNPs [23,45,46].

PCR and restriction products were resolved on 2% agarose gels (Amresco, Solon, OH, USA). All gels were stained with a nucleic acid gel stain (GelRed™, Biotium Inc, Hayward, CA, USA) and visualized under UV transillumination (GelDoc®, Biorad, Hercules, CA, USA). PCR products were purified and sequenced commercially (Macrogen Inc, Seoul, Korea).

*Pfmdr1* copy numbers were determined using real time PCR (ABI Prism® 7000 Sequence Detection System). Real time PCR reactions were run in triplicate for each sample as previously described [47]. *P. falciparum* strains 3D7, D10 and K1 with single copies of the *pfmdr1* gene were used as calibrators and FCB and Dd2 strains with multiple copies of the gene were used as controls. The sample copy numbers were calculated using a comparative threshold method (ΔΔCT). Assays were repeated if the following results were obtained: copy number 1.3-1.6 and 2.3-2.6 or CT value >35 or standard deviation value >0.5. Samples with repeated CT values >35 were considered to have failed.

### Statistical analyses

The exact incidence of *P. falciparum* malaria was not known and we therefore decided to collect as many samples as possible over a two year period. SNP frequencies were calculated by dividing the number of SNPs by the number of patients in whom a certain the allele could be identified. Allele frequencies in 2009 and 2010 were compared using Chi-squared tests.

### Ethics

Patients with uncomplicated malaria were enrolled and samples collected after informed oral consent of the patient or in the case of minors informed proxy-consent of their parent or guardian. The study was approved by the Institutional Ethics Committee of R D Gardi Medical College in Ujjain, Madhya Pradesh, India (61/2009) and the Regional Ethics Committee in Stockholm, Sweden (2011/832-32/2).

## Results

During the peak malaria seasons 2009 and 2010, 44 and 43 (total 87) patients with microscopically verified *P. falciparum* mono-infection were identified and included. The median age was 30 years (inter quartile range 15–30 years). Forty-four patients were male and 34 female, for the remaining nine individuals sex was not recorded. PCR was successful for at least one allele in all 87 samples. There were no significant differences in SNP frequencies between 2009 and 2010.

### *Pfdhfr* and *pfdhps*

The number of resistance-associated SNPs and haplotypes are shown in Tables 1 and 2. The resistance-associated *pfdhfr* 108N and 59R alleles were found in 75/78 (96%) 70/78 (90%) samples, respectively. No sample had resistance-associated SNPs at codons 16, 50, 51 or 164. *Pfdhfr* 108N and 59R alleles were found together in 70/78 (90%) of samples.

**Table 1:** **The number and frequency of resistance-associated *pfdhfr *** **and *pfdhps *** **alleles in *Plasmodium falciparum *** **samples collected Ujjain, Madhya Pradesh, India in 2009 and 2010**

***Pfdhfr***	**A16V**	**N51I**	**C59R**	**S108N**	**I164L**
**Sensitive**	69/69	78/78	8/78 (10%)	3/78 (4%)	78/78
**Resistant**	0/69	0/78	70/78 (90%)	75/78 (96%)	0/78
***Pfdhps***	**S436F**	**A437G**	**K540E**	**A581G**	**A613T**
**Sensitive**	0/76	70/77 (91%)	77/77	77/77	77/77
**Resistant**	76/76	7/77 (9%)	0/77	0/77	0/77

**Table 2:** **The number and frequency of *pfdhfr *** **and *pfdhps *** **resistance-associated haplotypes in *Plasmodium falciparum *** **samples collected in Ujjain, Madhya Pradesh, India in 2009 and 2010**

**No of each haplotype**	No SNPs*	***Pfdhfr***				***Pfdhps***				
		**51**	**59**	**108**	**164**	**436**	**437**	**540**	**581**	**613**
3 (4%)	1	N	C	S	I	***F***	A	K	A	A
4 (5%)	2	N	C	***N***	I	***F***	A	K	A	A
1 (1%)	3	N	C	***N***	I	***F***	***G***	K	A	A
62 (82%)	3	N	***R***	***N***	I	***F***	A	K	A	A
6 (8%)	4	N	***R***	***N***	I	***F***	***G***	K	A	A
2	2	N	***R***	***N***	I					
1	1					***F***	A	K	A	A

Resistance associated alleles are written in bold and are italics.

*Number of resistance-associated SNPs in each haplotype.

Haplotype proportions were calculated from the total number (76) of samples in which both *pfdhfr* and *pfdhps* haplotypes were identified.

The resistance-associated *pfdhps* 437G allele was found in 7/77 (9%) samples. No resistance-associated alleles were detected at codons 540, 581 or 613. All samples (76/76) carried the resistance-associated 436F allele.

Combined *pfdhfr* and *pfdhps* haplotypes were identified in 76 samples. Double mutant *pfdhfr* 59R + 108N were found in 62/76 (82%) samples and double mutant *pfdhfr* 108N and *pfdhps* 437G was found in 1/76 (1%) samples. Triple mutant *pfdhfr* 59R + 108 N and *pfdhps* 437G were found in 6/76 (8%) samples. All these haplotypes also had the 436 F allele potentially adding one resistance-associated SNP to each haplotype.

### Pfcrt

*Pfcrt* 76T was found in 82/87 (94%) and *pfcrt* K76 was found in 5/87 (6%) of samples. The *pfcrt* 72–76 haplotypes found were: 80/84 (95%) SVMNT, 3/84 (4%) CVMNK and 1/84 (1%) CVMNT. Sequencing failed altogether in three samples and identified only the *pfcrt* 72–75 haplotype in 13 samples. In these 13 samples, the 76 allele was detected using PCR-RFLP (Table 3).

**Table 3:** **The number and frequency of *pfcrt*, *pfmdr1 *** **and *pfnhe *** **ms 4760 alleles in *Plasmodium falciparum *** **field samples collected in Ujjain, Madhya Pradesh, India in 2009 and 2010**

**Gene**	**Polymorphism/no of repeats**	Number/total*	**Proportion (%)**
***Pfcrt *** **76**	K	5/87	6
	T	82/87	94
***Pfmdr1***			
**86**	N	70/83	84
**86**	Y	13/83	16
**1034 + 1042**	SN	75/75	100
**1246**	D	70/72	97
**1246**	Y	2/72	3
**Gene copy number†**	1	74/75	99
	2	1/75	1
***Pfnhe *** **ms4760 DNNND repeats**	1	14/74	19
	2	50/74	68
	3	3/74	4
	4	7/74	9
***Pfnhe *** **ms4760 DDNHNDNHNND Repeats**	1	56/74	76
	2	18/74	24

* Total number of polymorphisms successfully identified at each locus.

†*Pfmdr1* gene copy number was between one and two (ΔΔCt 1.3-1.6) in six samples and PCRs failed in six samples.

All *pfmdr1* 86Y occurred together with *pfcrt* 76 T. *Pfmdr1* 1246Y alleles occurred together with *pfcrt* 76 T and *pfmdr1* N86.

### Pfmdr1

*Pfmdr1* N86 and 86Y were identified in 70/83 (84%) and 13/83 (16%) samples, respectively. *Pfmdr1* 86Y only occurred together with *pfcrt* 76 T. *Pfmdr1* S1034 + N1042 were identified together in all 75 successfully sequenced samples. *Pfmdr1* D1246 and 1246Y were identified in 70/72 (97%) and 2/72 (3%) samples, respectively. No sample had the *pfmdr1* 1226Y allele associated with reduced sensitivity to artemisinin, lumefantrine and mefloquine [48].

One *pfmdr1* gene copy was found in 74/75 (99%) successfully amplified samples. After repeating PCRs three times, one sample had two *pfmdr1* copies and six samples had an indeterminate number of copies with ΔΔCt (cycle threshold) values consistently between 1.3 and 1.6. PCRs failed in six samples primarily due to CT values above the 35-cycle cut-off. Further details are shown in Table 3.

### Pfnhe1

The numbers of DNNND and DDNHNDNHNND repeats in *pfnhe1* ms4760 were identified in 74/87 (85%) samples (Table 3). Five previously described *pfnhe1* ms4760 profiles were detected at the following frequencies: ms4760-1 (n = 4), ms4760-2 (n = 14), ms4760-5 (n = 7), ms4760-6 (n = 46), ms4760-7 (n = 3). More than one DNNND repeat was found in 60/74 (81%) samples. The numbers of DDNHNDNHNND repeats were one in 56/74 (76%) and two in 18/74 (24%) of samples. The numbers of repeats were not associated with any SNP or haplotypes in *pfcrt*, *pfmdr1*, *pfdhfr* or *pfdhps*.

## Discussion

This is the first characterization of key anti-malarial drug resistance associated genetic polymorphisms among *P. falciparum* field isolates in Ujjain, Madhya Pradesh, central India. Importantly, this study was conducted prior to implementation of artemisinin-based combination therapy (ACT) in Ujjain and thus provides baseline data on the prevalence of resistance-associated polymorphisms prior to the introduction of ASP. From these data the prevalence of *in vivo* CQ and SP resistance in the study area can be inferred. The data also provide baseline information for future temporal surveillance of resistance-associated genetic polymorphisms in the study area as well as for comparisons with neighbouring areas.

Despite this study being conducted prior to the introduction of ASP, the SP resistance-associated 436F allele was found in all samples. This is an allele that has previously been described in several Indian states and that has been found to modulate SP susceptibility [21]. Typically, SP resistance arises through sequential selection of SNPs, starting with *pfdhfr* 108 N and 59R followed by SNPs in *pfdhps*[15]. It is thus most likely that 436F is a wild type allele in Ujjain. Nevertheless, it may well result in a degree of reduced susceptibility to SP [12].

The majority (82%) of samples in this study carried the SP resistance-associated double *pfdhfr* 59R and 108N together with *pfdhps* 436F. This suggests a moderate degree of reduced susceptibility to SP and a low risk of treatment failure at the time of the study. Furthermore, only one sample had two *pfmdr1* gene copies that have been linked to reduced artemisinin susceptibility. Based on these findings, it is likely that ASP is highly efficacious for treatment of uncomplicated *P. falciparum* malaria in Ujjain. However, four samples had *pfdhfr* 59R + 108N and *pfdhps* 436F + 437G indicating that these parasite subpopulations only need to acquire one more SNP in *pfdhfr* to become similar to *P. falciparum* that was associated with treatment failure in nearby West Bengal (*pfdhfr* 51I + 59R + 108N and *pfdhps* 437G or 436A + 540E) [8]. This is particularly concerning considering that this report is based on data collected before ASP was introduced in Ujjain.

Of even greater concern, previous experience suggests that SP resistance can and will spread rapidly despite the concurrent use of artesunate. Specifically, the prevalence of the quintuple DHFR + DHPS haplotype increased from 11 to 75% after only four years of using ASP in Mozambique [49]. Furthermore, CQ resistance swept across India and SP resistance spread through Southeast Asia in the past [50]. The existence of highly SP-resistant *P. falciparum* on the Andaman and Nicobar islands as well as in north-eastern India is, therefore, worrisome [18-21]. Spread of SP resistance will in turn expose artesunate to a high selective pressure, which may spur development and spread of new foci of decreased susceptibility to artemisinin. This highlights the need for continual monitoring of drug resistance in Ujjain as well as the rest of India.

The 95% prevalence of *pfcrt* 76T in Ujjain is similar to data from many other parts of India as well as neighbouring countries such as Pakistan [51,52]. The high prevalence suggests that treatment failure rates following intake of a total dose of 25 mg/kg of CQ are likely to be similar to the ~70% treatment failure rate reported from Goa in 2011, when the 76T prevalence was 100% [53]. In view of the high prevalence of *pfcrt* 76T, replacing CQ was undoubtedly the correct decision also for Ujjain. Sequencing of the *pfcrt* 72–76 haplotype identified the typical CQ “wild type” CVMNK haplotype associated with CQ sensitivity in the three patients that had K76. In patients harbouring the 76T allele, the principle haplotype found was SVMNT. This is the most common haplotype in India as well as Pakistan, Iran and probably originates from Papua New Guinea [17,51,52,54]. In addition, haplotype CVMNT that has also been described previously in India, was found [53]. Thus the *pfcrt* SNPs found in Ujjain are the same as in most of India in line with previous studies showing how CQ resistance spread throughout the country.

Given the proximity and similar genetic background of CQ-resistant *P. falciparum* in India and Pakistan it is likely that the genetic mechanism causing resistance is similar. It is therefore noteworthy that changing the CQ dosing schedule to 40 mg/kg as divided doses over five days compared to the standard 25 mg/kg over three days approximately doubled the efficacy of CQ (49 *vs* 26%) in Pakistan [52]. Although 49% efficacy is unacceptably low, this does suggest that CQ resistance is dose dependent in *P. falciparum* with *pfcrt* 72–76 SVMNT. Similarly, treatment with 50 mg/kg compared to 25 mg/kg over three days approximately doubled the efficacy (78 *vs* 38%) of CQ in *P. falciparum* with *pfcrt* 72–76 CVIET in West Africa [55]. It may therefore be worth considering whether increasing the dose and treatment duration of CQ can improve the efficacy of CQ in India. This is potentially interesting as considerably higher CQ doses given for longer periods can be well tolerated [56,57]. However, it is not known to what degree efficacy could potentially be improved with a higher dose regimen.

The finding of only one *pfmdr1* gene copy number in all but one sample indicates that *P. falciparum* in Ujjain do not presently have reduced susceptibility to artemisnin derivatives, lumefantrine, piperaquine or mefloquine [31]. However, in transfection experiments *pfmdr1* alleles S1034, N1042 and D1246 have been linked to reduced mefloquine and artemisinin sensitivity [29]. Thus the *P. falciparum* population in Ujjain have *pfmdr1* alleles that possibly are linked to reduced artemisinin sensitivity. The lack of *pfmdr1* amplifications and high prevalence of *pfcrt* 76 T suggests that artemether-lumefantrine would be efficacious in Ujjain.

The *pfnhe1* ms4760-6 haplotype that has two DNNND repeats and one DDNHNDNHNND repeat was predominant in Ujjain. In other studies in Asia the ms4760-7 haplotype that has three DNNND repeats and one NHNDNHNNDDD repeat has been the most prevalent [38,39,58]. Both these profiles have been found to have similarly reduced quinine sensitivity, suggesting that *P. falciparum* with a degree of reduced quinine sensitivity is prevalent in Ujjain [39]. However, the *pfmdr1* 86, 1034, 1042 and 1246 NSND haplotype that was highly prevalent in Ujjain has been associated with quinine sensitivity [29,59]. Also, none of the above is a marker of quinine treatment failure and the correlation with quinine susceptibility is not fully elucidated. Thus, available data are difficult to interpret but provide a baseline for future monitoring.

The principle limitation of this study is the relatively small sample size. However, there was no obvious bias in sample collection and the data are therefore probably representative of the study area. There is also a lack local knowledge about the correlation between the various molecular markers studied and reduced drug susceptibility. However, the principle CQ and SP resistance markers studied have been verified in clinical studies in India and found to be valid.

## Conclusions

This is the first report on anti-malarial resistance-associated SNPs among *P. falciparum* field isolates in Ujjain, Madhya Pradesh, central India, collected prior to the introduction of ASP. The results indicate that the efficacy of standard dose CQ was likely to be poor whereas ASP was likely to be efficacious supporting the shift from CQ to ASP as first line anti-malarial drug for uncomplicated *P. falciparum* malaria. However, *P. falciparum* with reduced susceptibility to SP is highly prevalent, highlighting the need for continuous surveillance of ASP efficacy.

## Abbreviations

ASP: Artesunate + sulphadoxine-pyrimethamine; CQ: Chloroquine; SNP: Single nucleotide polymorphisms; pfdhfr: *P. falciparum* dihydrofolate reductase gene; pfdhps: *P. falciparum* dihydropteroate synthase gene; pfcrt: *P. falciparum* chloroquine resistance transporter gene; pfmdr1: *P. falciparum* multidrug resistance gene 1; pfnhe1: *P. falciparum* sodium/hydrogen exchanger gene and ∆∆Ct, difference in cycle threshold.

## Competing interests

The authors have declared that they have no competing interests.

## Authors’ contributions

AP, AM and JU conceived the study. AP organized data collection. AP, AM, SG, JM, AS, VD, and JU designed the methodology. AP, SG, JM, AS, and VD collected the field samples. AP, AM and JU conducted the molecular analyses. AP, JU and AM drafted the manuscript. SBG, JM, AS, and VD provided valuable insights during the revision and editing of the manuscript. All authors read and approved the final manuscript.
